# Silibinin‐Loaded Nanoparticles for Drug Delivery in Gastric Cancer: In Vitro Modulating miR‐181a and miR‐34a to Inhibit Cancer Cell Growth and Migration

**DOI:** 10.1002/fsn3.4609

**Published:** 2025-03-11

**Authors:** Mohsen Karami Fath, Parastoo Vakilinezami, Zohre Abdoli Keleshtery, Zahra Sima Azgomi, Sharareh Nezamivand Chegini, Mahdi Shahriarinour, Saman Seyfizadeh Saraabestani, Mahzad Diyarkojouri, Mohammad Nikpassand, Najmeh Ranji

**Affiliations:** ^1^ Department of Cellular and Molecular Biology, Faculty of Biological Sciences Kharazmi University Tehran Iran; ^2^ Department of Biology, Faculty of Basic Sciences, Rasht Branch Islamic Azad University Rasht Iran; ^3^ Department of Biology Payame Noor University (PNU) Tehran Iran; ^4^ Department of Chemistry, Faculty of Basic Sciences, Rasht Branch Islamic Azad University Rasht Iran

**Keywords:** antimetastatic, micell/liposome, microRNAs, silibinin, wound healing

## Abstract

Silibinin (C_25_H_22_O_10_), a notable bioactive flavonolignans, is recognized for its anticancer properties. However, due to its poor water solubility, the objective of this study was to design and synthesize nanocarriers to enhance the solubility of silibinin for effective delivery to AGS gastric cancer cells. This study details the synthesis of PEG_400_‐OA nanoparticles for silibinin delivery to AGS cells. Various physicochemical techniques, including FT‐IR, TGA, EDX, FE‐SEM, and TEM, were employed to characterize the silibinin‐loaded nanoparticles (SLNs), confirming particle size, elemental composition, thermal stability, and paramagnetic properties. The anticancer effects of the SLNs were assessed using MTT assay, scratch test, and Q‐RT‐PCR. The SLNs exhibited particle sizes ranging from 45 to 60 nm, with thermal stability below 110°C. TEM images suggested a micelles/liposomes structure due to the low polydispersity and spherical shape of the particles. EDX analysis revealed the presence of C, O, N, and P, confirming the incorporation of phospholipids (micelle/liposome) within the SLNs. The IC_50_ of SLNs in AGS cells was determined to be 28.21 μg/mL. Antimigration effects of SLNs's were demonstrated through the downregulation of miR‐181a and upregulation of its potential targets (*TGFB*, *SMAD3*, and *β‐catenin* genes), as well as the upregulation of miR‐34a and downregulation of its potential target (*E‐Cadherin* antimigration gene). The findings suggest that nanoparticles serve as effective nanocarriers for the targeted delivery of silibinin to cancer cells. Silibinin‐loaded micelles/liposomes nanoparticles (SLNs) appear to inhibit cancer cell proliferation and migration by modulating the expressionof miRNAs and their target mRNAs.

## Graphical abstract

Silibinin nanoparticles significantly modulate miR‐181a and miR‐34a. This modulation leads to reduced cell migration and invasion. The results suggest a potential therapeutic strategy for gastric cancer.

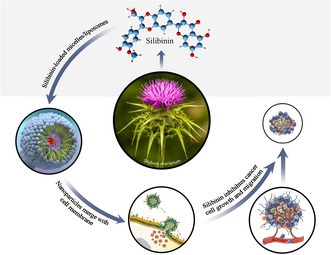

## 1 Introduction

Silibinin (C_25_H_22_O_10_) (Deep and Agarwal 2010) is the most important bioactive component (flavonolignans) extracted from *Silybum marianum* (Zhong et al. 2015; Maleki Zadeh et al. 2016). Silymarin derived from *Silybum marianum* exhibits hepatoprotective (Nikzad et al. 2017), anticarcinogen (Deep and Agarwal 2010), and immunostimulatory (Wilasrusmee et al. 2002) properties. It has been found to display diverse therapeutic effects, functioning as a potential remedy for various cancers, including lung, prostatic, colon and breast cancers. These effects are mediated through the regulation of cell proliferation, apoptosis (programmed cell death), angiogenesis, and other cellular mechanisms (Zhu et al. 2016). Despite the therapeutic potential of silibinin and *Silybum marianum* , their low solubility in water significantly reduces their bioavailability after administration. A study involving six healthy subjects reported maximum serum concentrations of silibinin between 0.18–0.62 μg/mL following a 240 mg dose (Bijak 2017). Therefore, researchers are actively pursuing new strategies to enhance the solubility of silibinin and other water‐insoluble therapeutic agents.

MicroRNAs (miRNAs) are short non‐coding RNAs that regulate gene expression by degrading mRNAs or inhibiting their translation (Svoronos, Engelman, and Slack 2016). It is estimated that more than 60% of all human genes are regulated by over 2588 miRNAs (Shu et al. 2017). miRNAs play critical roles in various biological processes, such as cell proliferation (Pakizehkar et al. 2020a), migration (Wang et al. 2022), and apoptosis (Pakizehkar et al. 2020a). Abnormal miRNA expression is a key feature in many cancers (Faramin Lashkarian et al. 2023), where they can act as oncogenic or tumor suppressors (Fattahi et al. 2023). For example, miR‐34a is a tumor‐suppressive miRNA that inhibit cell cycle progression, invasion, and metastasis (Hwang et al. 2023). It suppresses *Akt* expression in the PI3K/Akt signaling pathway, reducing cell proliferation. Su et al. (2023) demonstrated that miR‐34a inhibits malignancy in oral cancer cells by regulating the Axl/Akt/GSK‐3β pathway, which affects downstream genes such as *cyclin D1*, *β‐catenin*, and *c‐Myc*.

miR‐181a has a dual function, acting as an onco‐miR in some cancers, such as stomach (Lu et al. 2018), and breast cancers (Tian et al. 2018), while functioning as a tumor suppressor in others, such as glioma (Shi et al. 2008). In colon cancer, miR‐181a promotes proliferation and invasion by targeting the tumor suppressor gene *PTEN* (Li, Shen, et al. 2023), while in colorectal cancer, miR‐181a‐5p induces G0/G1 cell cycle arrest and inhibits invasion and migration (Yu et al. 2019).

Invasion and metastasis are key hallmarks of cancer, primarily driven by the epithelial–mesenchymal transition (EMT) process (Hwang et al. 2023). EMT is characterized by morphological changes, loss of cell polarity, reduced cell adhesions, and increased migration, all of which promote tumor invasion and metastasis (Li, Xia, et al. 2023). Transforming growth factor‐β (TGF‐β) signaling is a major stimulator of EMT and plays a crucial role in tumor progression and metastasis. TGF‐β exhibits dual functionality, initially acting as a tumor suppressor in early cancer stages and later as a cancer promoter during progression (Wang and Zhou 2011). Upon TGF‐β/TGF‐β binding to its receptor, Smad2 and Smad3 are activated, which enhances EMT in mammary epithelial cells (Valcourt et al. 2005). E‐cadherin, located in the cell membrane, binds to β‐catenin to maintain cellular adhesion and inhibit EMT, invasion, and metastasis. Increased nuclear β‐catenin and decreased E‐cadherin levels, along with reduced membrane‐bound β‐catenin, are commonly observed in various cancer types (Valcourt et al. 2005).

Metastasis remains a significant challenge in cancer treatment, accounting for a major causes of treatment failure and cancer‐related deaths (Kilmister et al. 2022). Cancer stem cells are thought to play a critical role in relapse, chemotherapy resistance, and metastasis (Stuelten, Parent, and Montell 2018). Effectively targeting cancer stem cells, along with inhibiting cancer migration and metastasis, is a critical step in combating cancer (Li et al. 2021). Developing new therapeutic agent that can effectively target all cancer cells, particularly cancer stem cells, while inhibiting migration and metastasis, is an essential step in combating cancer. Migrastatic therapeutic strategies, which specifically inhibit cancer cell migration and invasion through the extracellular matrix (ECM), represent a promising approach (Raudenská et al. 2023).

However, the low solubility of many pharmaceutical agents presents an ongoing challenge for drug formulation, often leading to poor bioavailability after oral administration (Gao et al. 2020). To overcome this, researchers employ various nanostructures, including dendrimers, liposomes, nano‐emulsions, microemulsions, solid lipid nanoparticles, and polymersome, to improve the solubility of poorly soluble compounds (Pakizehkar et al. 2020b; Salunkhe et al. 2014).

In this study, we synthesized silibinin‐loaded micelle/liposome nanoparticles (SLNs). After conducting physicochemical analysis, we evaluated the anticancer and migrastatic properties of SLNs in AGS gastric cancer cells. To further explore the roles of miRNAs in regulating target genes, we assessed the effects of silibinin‐loaded SLNs on the expression of miR‐181a, miR‐34a, and their potential targets in cell migration, adhesion, and apoptotic pathways.

## 2 Materials and Methods

### 2.1 Materials

Oleoyl chloride (> 89%), polyethylene glycol 400 kD, and triethylamine were obtained from Sigma‐Aldrich Co. (Saint Louis, MO, USA). Silibinin (> 98% purity), phosphate buffer saline (PBS), and chloroform were purchased from Merck Co. (Darmstadt, Germany). All solvents used were of analytical grade, with no further chemical modification.

### 2.2 Synthesis of Nanoparticles

Polyethylene glycol (PEG_400_)‐oleic acid (OA) nanoparticles were synthesized following the esterification reaction method described by (Tahmasebi Mirgani et al. 2014). Briefly, 3.01 g of oleoyl chloride (0.01 mol) was added to 20 g of PEG_400_ (0.01 mol) and triethylamine (1.2 g, 0.012 mol) in 5 mL of chloroform solvent. The mixture was stirred at room temperature for 4 h. Triethylammonium chloride salt was removed using a 0.45 μm syringe filter (Merck Millipore, USA). The chloroform was evaporated in a vacuum oven at 40°C for 4 h.

### 2.3 Silibinin Loading in Nanoparticles

To load silibinin into the nanoparticles, 50 mg of silibinin dissolved in 5 mL of acetone was mixed with 300 mg of PEG_400_‐OA nanoparticles to achieve a 1:6 weight ratio. The mixture was stirrered for 4 h, during which silibinin completely dissolved in the nanoparticles without any precipitation. Afterward, the acetone was evaporated, and a 1 mg/mL concentration of silibinin was prepared in PBS to form micelle/liposome structure. To prevent degradation, the silibinin‐loaded nanoparticles were protected from light. Any unencapsulated silibinin was removed using a 0.22 μm syringe filter (Merck Millipore).

### 2.4 Physicochemical Properties

#### 2.4.1 Fourier Transform Infrared (FTIR) Analysis

The FT‐IR spectra of PEG_400_‐OA nanoparticles and silibinin‐loaded micelle/liposome nanoparticles (SLNs) were recorded using a Shimadzu FTIR‐8400S spectrometer (Shimadzu Europe). The spectra were measured over a range of 400–4000 cm^−1^.

#### 2.4.2 Thermal and Magnetic Characteristics of SLNs

The thermal characteristics of the silibinin‐loaded micelle/liposome nanoparticles (SLNs) were analyzed using a thermosgravimetric analyzer (TGA) (TA Q600, USA). Magnetic properties were examined using a vibrating sample magnetometer (VSM) (Lakeshore 7403, USA).

#### 2.4.3 Energy‐Dispersive X‐Ray (EDX) Spectrometry

The chemical composition of the silibinin‐loaded micelle/liposome nanoparticles (SLNs) was determined using energy‐dispersive X‐ray (EDX) spectrometry, performed with a VEGA\TESCAN‐LMU instrument (Czech Republic) for elemental point analysis.

#### 2.4.4 Transmission Electron Microscopy (TEM)

TEM was performed using a Philips CM 200 microscope operating at 200 kV to evaluate the size, morphology, and internal structures of the bare and silibinin‐loaded nanoparticles (SLNs). Samples were prepared by suspending SLNs in ethanol for 10 min in an ultrasonic bath. The sample was then diluted with ethanol, loaded onto carbon‐coated copper grids, and dried at room temperature before microscopic imaging.

#### 2.4.5 X‐Ray Diffraction (XRD) Analysis

Nanostructures were detected using a Philips Xpert X‐ray powder diffraction (XRD) diffractometer with CuKα, radiation (*λ* = 0.154056) at a scanning speed of 2°/min, covering a range from 10° to 80° (2θ).

### 2.5 *In Vitro* Analysis

#### 2.5.1 Determination of Cell Viability by MTT Assay

Gastric cancer cells (AGS) were obtained from the National Cell Bank of Iran (NCBI, Pasteur Institute of Iran). The cells were cultured in 96‐well tissue culture plates at a density of 5 × 10^3^ cells/well. AGS cells were treated with silibinin, SLNs, and free nanoparticles at varying concentrations (0–100 μg/mL). After 24, 48, and 72 h, the treated and untreated cells were washed with phosphate‐buffered saline (PBS). Fresh medium (100 μL) containing MTT dye (0.5 mg/mL) was added to each well, followed by incubation for 3 h at 37°C in a humidified atmosphere with 5% CO_2_. To dissolve the formazan crystals formed, 100 μL of DMSO was added to each well. The optical density (OD) of the treated and untreated cells was measured at 570 nm, with a reference wavelength of 630 nm, using a microplate reader (ELx800; BioTek, USA). Cytotoxicity of silibinin, SLNs and free nanoparticles was calculated using the following formula:

$$\text{cytotoxicity}\;\left(\%\right)=\frac{{\mathrm{OD}}_{\left(\text{control}\right)}-{\mathrm{OD}}_{\left(\text{treat}\right)}}{{\mathrm{OD}}_{\left(\text{control}\right)}}\times 100$$

#### 2.5.2 Wound Healing (Scratch) Assay

To evaluate in vitro two‐dimensional cell migration, AGS gastric carcinoma cells were seeded in a six‐well plate at the concentration of 1 × 10^6^ cells per well in complete medium containing 10% FBS overnight, achieving > 90% confluency. A scratch (1.0 mm) was created using a 100 μL sterile pipette tip. After washing away detached cells, the remaining monolayer was treated with SLNs at the IC_50_ concentration (test group), while untreated cells served as the control group. The scratched areas were visualized after 24 h using a Nikon's inverted microscope at 40× magnification.

#### 2.5.3 Programmed Cell Death Analysis with SLNs in AGS Cells

Programmed cell death (apoptosis) induction with silibnin‐loaded SLNs was analyzed using PI/AnnexnV staining with an Annexin V‐FITC kit (BioLegend, USA). To determine the percentage of apoptosis, 1 × 10^6^ cells were seeded into two 25 mL culture flasks and incubated for 24 h. The cultured cells were then treated with 0 (control) and 28.21 μg/mL of SLNs for 24 h. According to the manufacturer's instructions, Annexin V‐FITC and propidium iodide (PI) solution were added to the treated cells and analyzed using a flow cytometry instrument (BD FACS Calibur; BD Biosciences, San Jose, CA, USA).

#### 2.5.4 Expression Pattern of miR‐181a and miR‐34a

The expression of miR‐181a and miR‐34a was quantitatively measured using real‐time PCR. Total RNA was extracted from treated and untreated cells (28.21 μg/mL of SLNs) after 24 h using the RNX‐PlusTM kit (SinaClon, Tehran, Iran). cDNA synthesis was performed using the BON stem High Sensitivity microRNA 1st‐Strand cDNA Synthesis Kit (Stem Cell Technology Research Center, Tehran, Iran). Initially, total RNA and BON‐RT adaptor (stem‐loop primers, 1 μM) were incubated at 75°C for 5 min in a thermocycler (BioRad, USA). Then dNTPs, RT enzyme, and buffer were added, and cDNA synthesis was carried out at 37°C for 10 min, followed by 55°C for 60 min, and 70°C for 10 min.

Q‐RT‐PCR was conducted using Universal SYBR qPCR Master Mix (2x) (Stem Cell Technology Research Center) on a Rotor‐GeneQ instrument (QIAGEN). The PCR program consisted of an initial denaturation at 95°C for 5 min, followed by 40 cycles at 95°C for 5 s and 60°C for 5 s. The primers used are listed in Table 1. They were designed by the Stem Cell Technology Research Center, with SNORD47 serving as the endogenous control. The expression of miRNAs was evaluated using the 2^−ΔΔ*Ct*^ method.

**TABLE 1:** Sequence of miRNA primers used for Q‐RT‐PCR.

Primer name	Primer sequence
miR‐34a‐F	5′‐AGGGTGGCAGTGTCTTAG‐3′
miR‐181a‐F	5′‐ACCAACATTCAACGCTG‐3′
SNORD47	5′‐ATCACTGTAAAACCGTTC‐3′
miR universal‐R	5′‐GAGCAGGGTCCGAGGT‐3′

#### 2.5.5 Prediction of Putative Targets of miR‐181a and miR‐34a

The potential targets of miR‐181a and miR‐34a related to cell adhesion and migration were predicted through *in silico* analysis using several algorithms, including miRWalk (http://mirwalk.umm.uni‐heidelberg.de/), DIANA TOOLS (http://diana.imis.athena‐innovation.gr/DianaTools/index.php), and TargetScan (https://www.targetscan.org/vert_80/). These software tools utilize various parameters, such as complimentary binding sites for miRNAs in the 3′‐UTR of potential target mRNAs, and the thermodynamical stability of miRNA‐mRNA interactions.

#### 2.5.6 Expression Pattern of Putative Targets of miR‐181a and miR‐34a

The transcription levels of the potential targets of miR‐181a and miR‐34a, which are involved in cell adhesion and cell migration namely, *CDH1* (*E‐cadherin*), *CTNNB* (*B‐catenin*), *TGF‐β* (Transforming Growth Factor Beta), and *Smad3* (SMAD family member 3) were analyzed using Q‐RT‐PCR. Total RNA was isolated using the RNX‐Plus kit (SinaClon), and cDNA synthesis was performed using a cDNA synthesis kit (Yekta Tajhiz Azma). Quantitative real‐time PCR was carried out using YTA SYBR Green qPCR MasterMix 2X (Yekta Tajhiz Azma, Iran) on a Rotor‐Gene Q instrument (QIAGEN). The Q‐RT‐PCR program included an initial denaturation at 95°C for 3 min, followed by 40 cycles of 95°C for 5 s, 60°C for 5 s, and 75°C for 10 s. The primers used are listed in Table 2. Gene expression was normalized to *GAPDH*, which served as the endogenous control. The relative expression of the putative target mRNAs was calculated using the 2^−ΔΔ*Ct*^ method.

**TABLE 2:** Sequence of primers used for Q‐RT‐PCR.

Primer name	Primer sequence	Length of production	Reference
*CDH1*‐F	5′‐ACGCATTGCCACATACACTC‐3′	181 bp	In this study
*CDH1*‐R	5′‐CTCCATCACAGAGGTTCCTG‐3′		
*CTNNB*‐F	5′‐GACAATGGCTACTCAAGCTG‐3′	264 bp	In this study
*CTNNB*‐R	5′‐GCATACTGTCCATCAATATCAGC‐3′		
*TGFB*‐F	5′‐GACATCAACGGGTTCACTACC‐3′	318 bp	In this study
*TGFB*‐R	5′‐CAGGACCTTGCTGTACTGC‐3′		
*SMAD3*‐F	5′‐AGAGACACCAGTTCTACCTCC‐3′	222 bp	In this study
*SMAD3*‐R	5′‐GAACCTGCGTCCATGCTG‐3′		
*GAPDH*‐F	5′‐CTCAAGATCATCAGCAATGCCTCC‐3′	113 bp	In this study
*GAPDH*‐R	5′‐ATGGCATGGACTGTGGTCATGAG‐3′		

### 2.6 Statistical Analysis

The statistical significance of differences between treated and untreated cells was assessed using one‐way ANOVA, while differences in gene expression were evaluated using the student *t*‐test. Statistical analyses were conducted using Prism9.4.1 software (GraphPad Software Inc., LaJolla, CA, USA), with significance defined as *PM* < 0.05.

## 3 Results

### 3.1 Physical and Chemical Characterization of Silibinin‐Loaded Micelle/Liposome Nanoparticles (SLNs)

**FIGURE 1:**
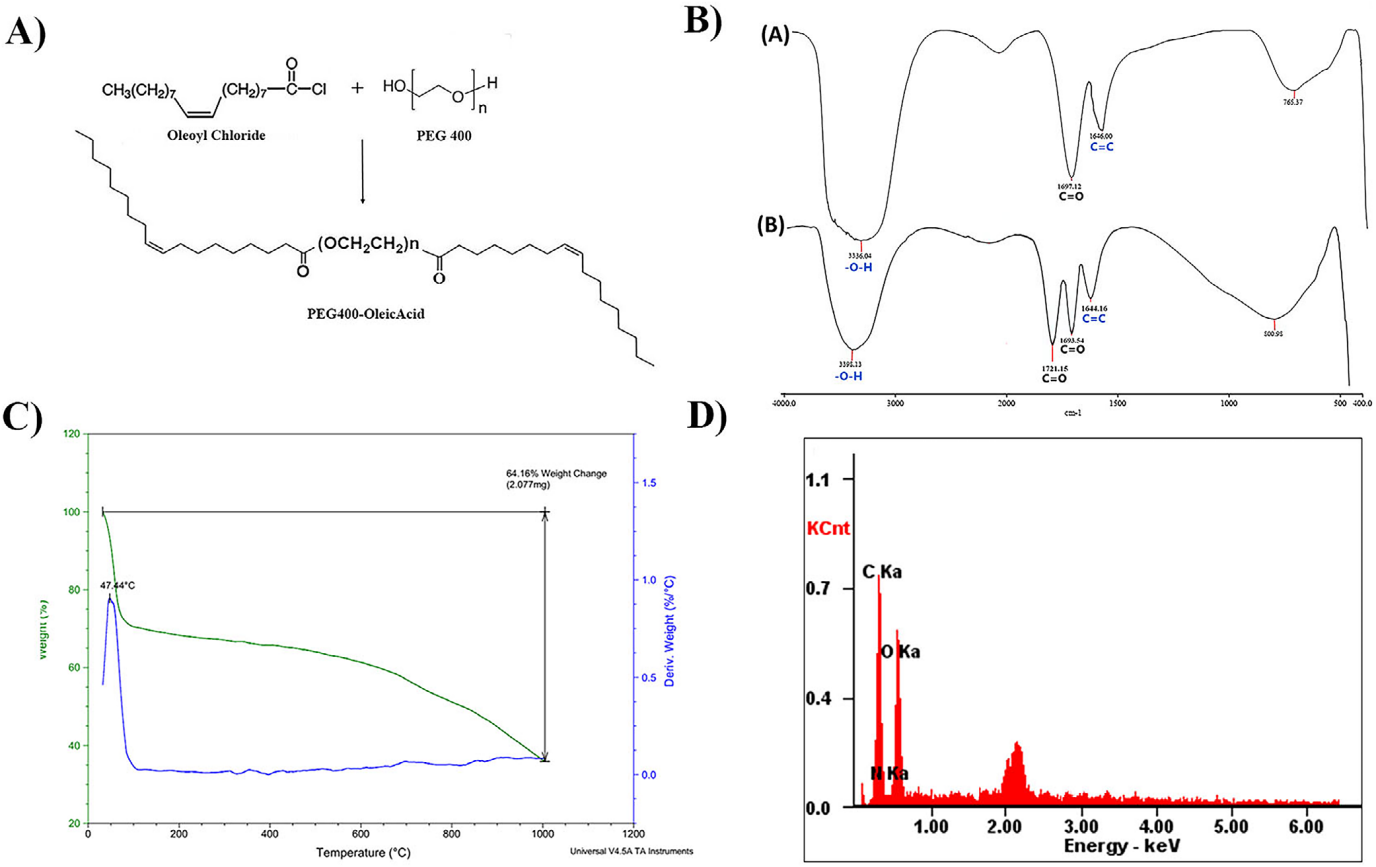
Physicochemical properties. (A) Molecular structure of the PEG_400_‐OA nanoparticle. (B) FT‐IR spectrum of the (A) free micelle/liposome nanoparticles and (B) silibinin‐loaded micelle/liposome nanoparticles (SLNs). (C) TGA‐DTG of silibinin‐loaded micelle/liposome nanoparticles (SLNs). (D) EDX of silibinin‐loaded micelle/liposome nanoparticles (SLNs).

The esterification reaction between oleoyl chloride and PEG_400_ in the organic phase was employed to prepare PEG_400_‐OA nanoparticles. The schematic representation of the reaction is illustrated in Figure 1A.

#### 3.1.1 FT‐IR Analysis

**FIGURE 2:**
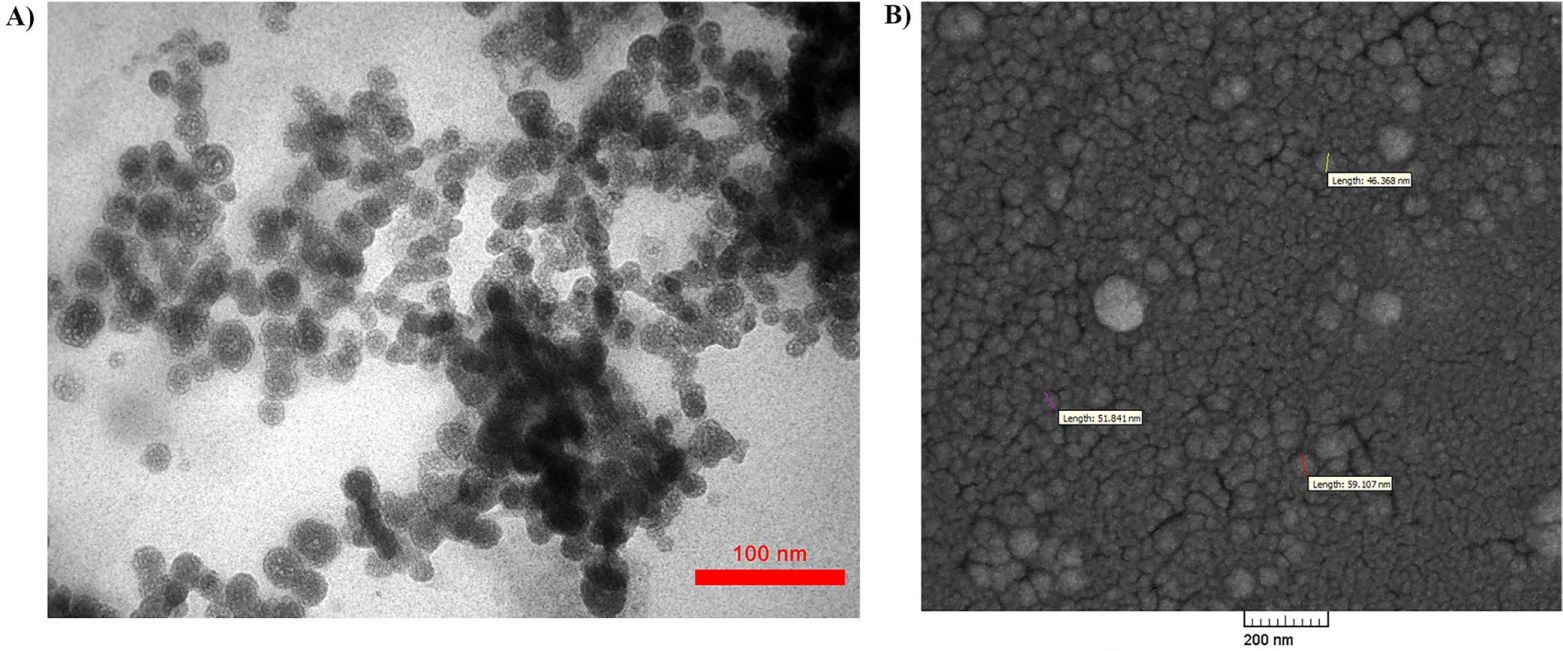
Morphology of SLNs. (A) TEM image of silibinin‐loaded micelle/liposome nanoparticles (SLNs). (B) FE‐SEM image of silibinin‐loaded micelle/liposome nanoparticles (SLNs).

FT‐IR spectroscopy confirmed the synthesis of PEG_400_‐OA (Figure 2). The nanoparticles incorporate the hydrophilic segments of polyethylene glycol and the hydrophobic segments of oleic acid. The FT‐IR spectrum of PEG_400_‐OA revealed broad stretching bands corresponding to the O–H group at around 3436 cm^−1^, while the C=O stretching band of the ester group appeared at 1697 cm^−1^, indicating the formation of a stable covalent bond between oleic acid and polyethylene glycol. So, C=C Bonds in PEG‐400 polymer appeared at 1646 cm^−1^. The FT‐IR spectrum of the SLNs structure showed bands related to both carbonyl ester from PEG_400_‐OA and the ketone part of silibinin at 1693 and 1721 cm^−1^, respectively, confirming the formation of SLNs. The presence of O–H and C=C bonds further indicated the formation of this structure (Figure 1B).

#### 3.1.2 TGA Analysis

**FIGURE 3:**
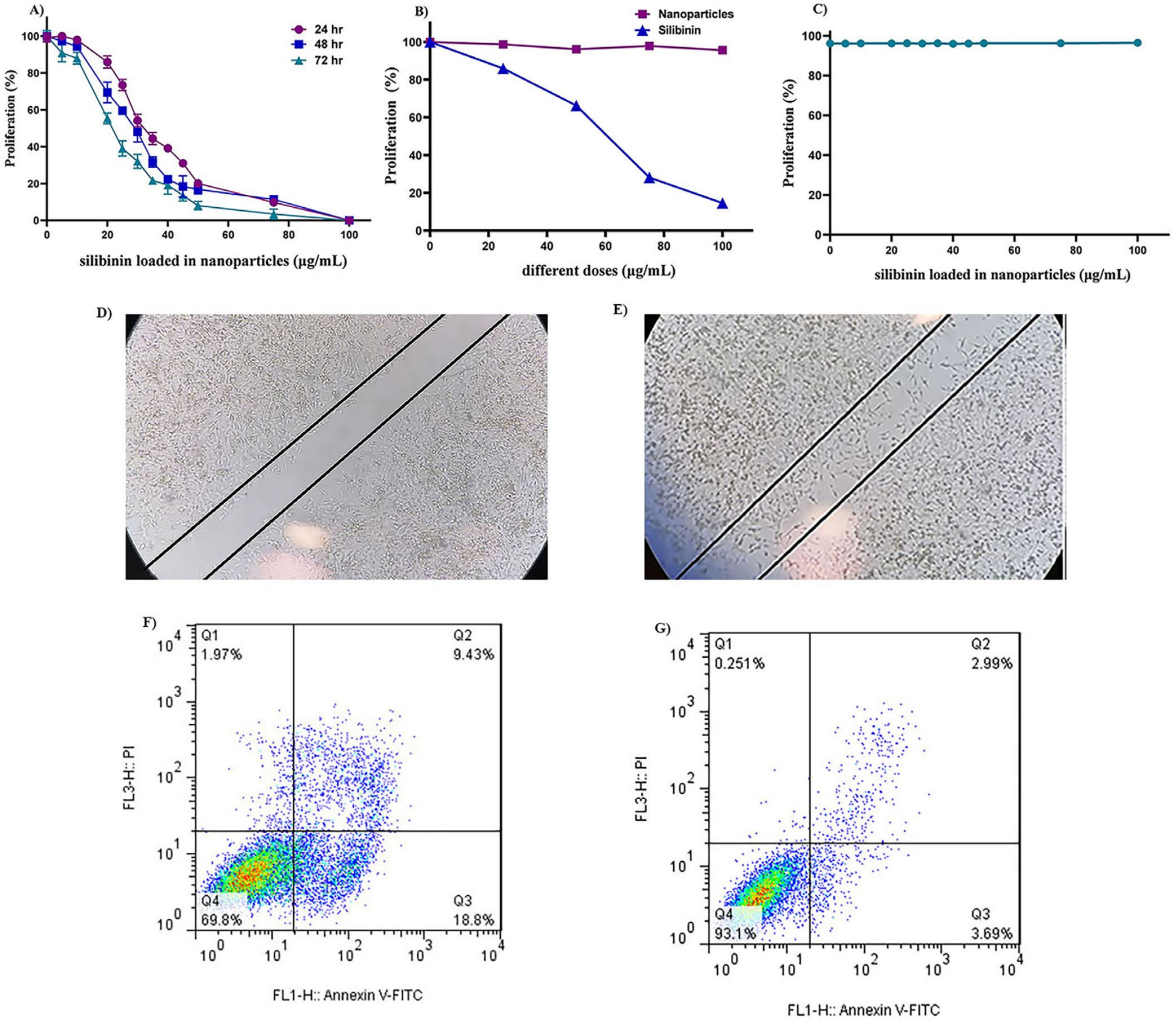
Effects of Silibinin‐loaded micelle/liposome nanoparticles on AGS gastric cancer cells and HFF_2_ normal cells. The effects of different concentrations (0–100 μg/mL) of (A) silibinin‐loaded micelle/liposome nanoparticles and (B) silibinin, and free micelle/liposome nanoparticles on the cell viability of AGS cells. (C) Viability of HFF_2_ normal cells treated with different doses of silibinin‐loaded micelle/liposome nanoparticles (0–100 μg/mL) for 24 h. (D) Suppression of AGS cell migration by silibinin‐loaded micelle/liposome nanoparticles (SLNs, 28.21 μg/mL) in compared to (E) Control (untreated cells) after 24 h (Magnification, 10×). (E) flow cytometry analysis of PI/annexinV staining on AGS cells (F) treated with IC_50_ of SLNs in compared to (G) untreated cells. Q1, Q2, Q3, and Q4 marked in each panel indicate the necrosis, late apoptosis, early apoptosis, and lived cells, respectively.

TGA measurement (TA Q600, USA) revealed a weight loss of approximately 27% (Figure 3) for PEG_400_‐OA at 47°C, due to the dehydroxylation of hydroxyl groups on the surface and the desorption of physically adsorbed water. These data indicate that the synthesized compound possesses good structural stability at room temperature and even body temperature, making it suitable for biological studies. However, it may not be ideal for industrial and laboratory use at higher temperatures (Figure 1C).

#### 3.1.3 Energy‐Dispersive X‐Ray Analysis

EDX analysis of the synthesized silibinin‐loaded micelle/liposome nanoparticles revealed the presence of C (59.63 w/w%), O (20.78 w/w%), N (7.21 w/w%), and P (12.38 w/w%), confirming the existence of phospholipids (micelle/liposome) within the nanoparticle structure (Figure 1D).

#### 3.1.4 TEM and SEM Images

TEM analysis was employed to examine the morphology and dimensions of the synthesized nanoparticles, revealing well‐dispersed particles with sizes ranging from 22 to 35 nm. The images demonstrated the uniformity of the particle components, displaying a symmetrical and systematicarrangement. Nanoparticles with low polydispersity and spherical shape resembling micelles/liposomes were observed (Figure 2A). Additionally, the FE‐SEM image revealed particles ranging from 45 to 60 nm is size, indicating homogeneity and a uniform, symmetrical distribution (Figure 2B).

### 3.2 *In Vitro* and *In Silico* Analysis of Anticancer Effects of SLNs

#### 3.2.1 Antiproliferative Effects of SLNs

**FIGURE 4:**
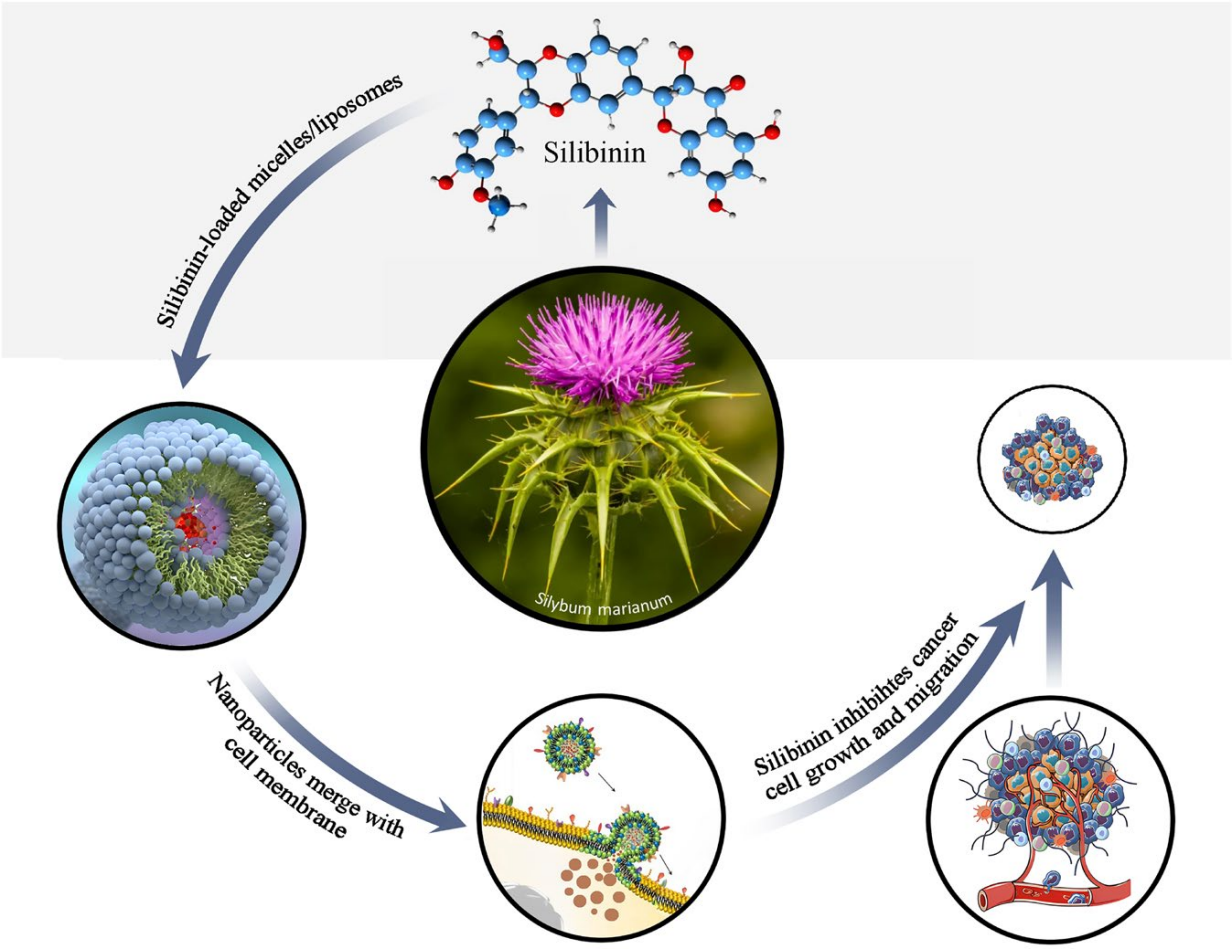
A schematic model of the effects of silibinin‐loaded micelle/liposome nanoparticles on cancer cell growth and migration. It seems that micelles and liposomes first merge with the cell membrane and then release their content (silibinin) into the cancer cells.

The viability of AGS cells treated with silibinin (0–100 μg/mL) and silibinin‐loaded micelle/liposome nanoparticles (SLNs, 0–100 μg/mL) was assessed using an MTT assay. The analysis revealed a dose‐ and time‐dependent decrease in cells viability. The half‐maximal inhibitory concentration (IC_50_) of SLNs was 28.21, 25, and 21.42 μg/mL after 24, 48, and 72 h, respectively (Figure 3A). According to a schematic model (Figure 4), silibinin molecules were loaded in micelle/liposome nanoparticles. Then, nanoparticles merged with the cancer cell membrane, released silibinin into the cancer cells and inhibited cell growth and migration.

In contrast, the IC_50_ of silibinin alone was 60.24 μg/mL after 24 h (Figure 3B). SLNs exhibited < 5% cytotoxicity in HFF2 normal cells after 24 h (Figure 3C). Additionally, micelle/liposome nanoparticles without silibinin exhibited < 5% cytotoxicity in AGS cells after 24 h (Figure 3B).

#### 3.2.2 *In Vitro* Anti Invasive Effect of Silibinin‐Loaded Micelles/Liposomes

The wound healing assay, used to measure in vitro cell migration (Lin et al. 2020) that silibinin‐loaded micelle/liposome nanoparticles suppressed the migration of AGS cancer cells at an IC_50_ concentration (28.21 μg/mL) after 24 h, compared to control cells (untreated cells) in vitro (Figure 3D,E).

#### 3.2.3 Apoptosis Induction by Silibinin (SLNs) in AGS Gastric Cancer Cells

Flow cytometry analysis of PI/Annexin V staining demonstrated that silibinin‐loaded micelle/liposome nanoparticles (SLNs, 28.21 μg/mL) induced apoptosis in AGS cells after 24 h. Early apoptosis (Annexin V^+^/PI^−^) increased to 18.8% in treated AGS cells, compared to 3.69%. in untreated cells. Late apoptosis (Annexin V^+^/PI^+^) also increased to 9.43% in treated cells, compared to 2.99% in untreated cells. Necrosis in SLNs‐treated cells was 1.97% compared to 0.251% in untreated cells (Figure 3F,G).

#### 3.2.4 Deregulation of miR‐181 and miR‐34a After Silibinin Induction

**FIGURE 5:**
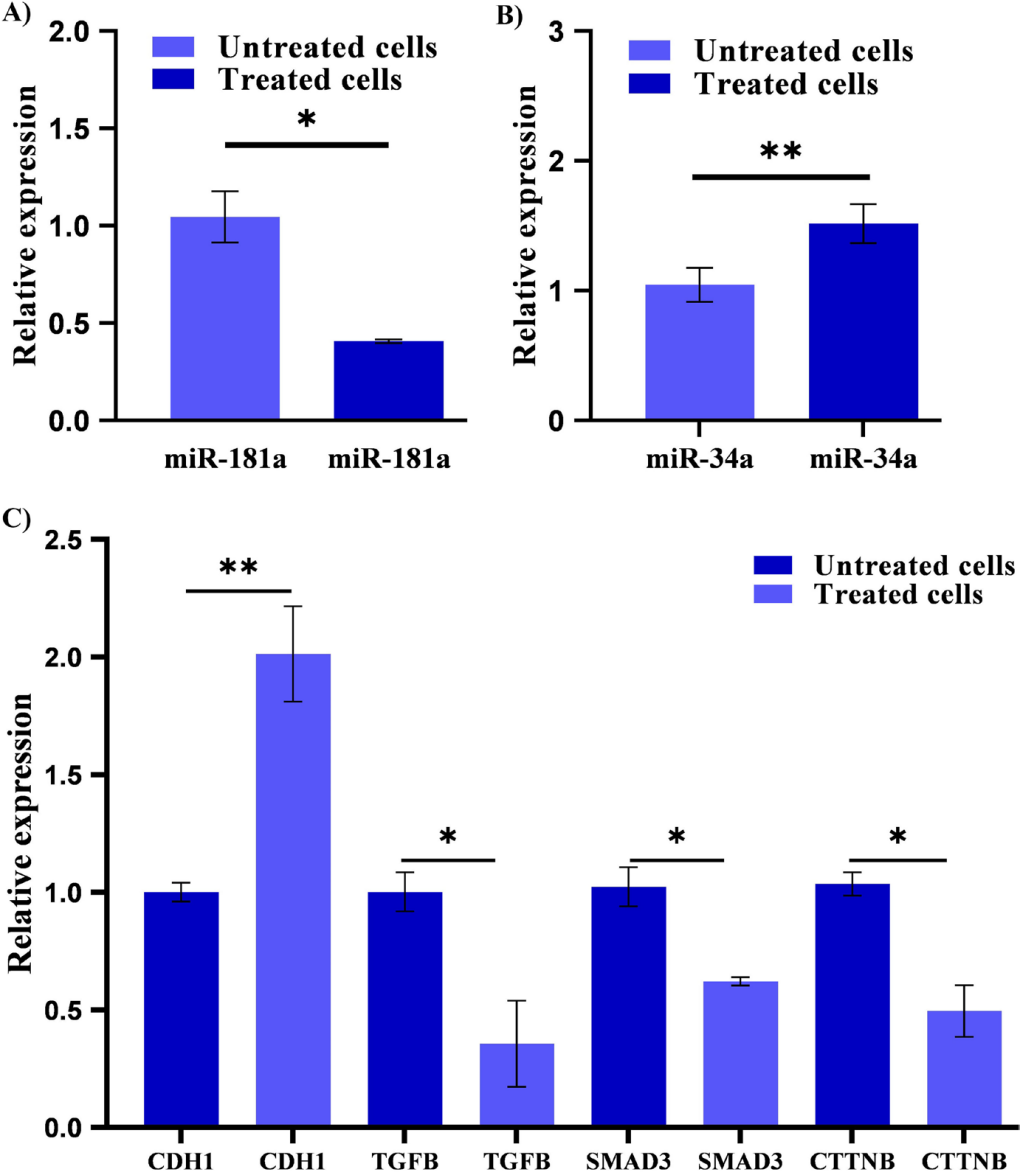
Relative expression of miRNAs and their potential targets. The quantitative expression of (A) miR‐181a and (B) miR‐34a, as well as (C) their potential targets, including *CDH1* (*E*‐*Cadherin*), *TGFB*, *SMAD3*, and *CTNNB* (*β‐catenin*) genes in AGS cancer cells treated with silibinin‐loaded micelle/liposome nanoparticles (28.21 μg/mL) and in untreated cells. Each experiment was conducted at least three times, with results represented as mean ± SD. Asterisks indicate significant differences between the treated and untreated cell groups (*p < 0.05, and **p < 0.01).

In AGS cancer cells treated with silibinin‐loaded micelle/liposome nanoparticles (28.21 μg/mL), we evaluated the expression levels of miR‐181a and miR‐34a. Quantitative RT‐PCR analysis revealed that the tumor‐suppressive miR‐34a was upregulated by 1.51 ± 0.15 fold in the silibinin‐treated cells compared to the control (untreated cells). Additionally, the expression of the oncogenic miR‐181a (onco‐miR) was downregulated by 0.41 ± 0.01 folds (Figure 5A,B).

#### 3.2.5 Deregulation of Putative Targets of miRNAs

Bioinformatics analysis predicted potential targets of miR‐181a and miR‐34a related to cell migration and adhesion (Table 3). The expression levels of four predicted targets of these miRNAs in these pathways were quantitatively measured in cells treated with silibinin‐loaded nanoparticles and untreated cells. The expression level of *TGFB*, *SMAD3*, and *β‐catenin* (*CTNNB1*), which are involved in cell migration, were downregulated as potential targets of upregulated miR‐34a in silibinin‐treated (28.21 μg/mL) cells compared to untreated cells. Additionally, the expression of *E‐Cadherin* (involved in cell adhesion) significantly increased as a potential targets of downregulated miR‐181a following treatment with silibinin (Figure 5C).

**TABLE 3:** Some potential targets of miRNAs related to cell migration, and cell adhesion.

miRNA	Potential target	Gene name
	In the cell migration pathway	
miR‐34a	*CTNNB1*	Catenin beta 1
	*CTNND1*	Catenin delta 1
	*CTNND2*	Catenin delta 2
	*EGFR*	Epidermal growth factor receptor
	*TGFB*	Transforming growth factor beta
	*SMAD4*	SMAD family member 4
	*MMP2*	Matrix metallopeptidase 2
	*MMP14*	Matrix metallopeptidase 14
	In the cell adhesion pathway Zhong et al. (2015)	
miR‐181a	*CDH1*	E‐cadherin
	*ITGB8*	Integrin beta 8
	*CNTN4*	Contactin 4
	*EPHA4*	EPH receptor A4
	*L1CAM*	L1 cell adhesion molecule
	*PCDHA1*	Protocadherin alpha 1
	*PCDHA2*	Protocadherin alpha 2
	*PCDHA3*	Protocadherin alpha 3
	*PCDHA4*	Protocadherin alpha 4
	*PCDHA10*	Protocadherin alpha 10

## 4 Discussion

Silibinin, the major component of silymarin extracted from milk thistle (*Sylibum marianum*) (Deep and Agarwal 2010), exhibits hepatoprotective (Kostek et al. 2012), antioxidant (Haddad et al. 2011), and anticancer properties (Mashhadi Akbar Boojar, and Golmohammad 2020). To address the challenges of silibinin's low solubility and poor bioavailability, we designed and synthesized silibinin‐loaded micelle/liposome nanoparticles (SLNs). Subsequently, we then evaluated the antimigration, antiproliferation and apoptotic effects of these silibinin‐loaded micelle/liposome nanoparticles on AGS gastric cancer cells.

Silibinin's poor bioavailability is due to its multi‐ring structure, which makes it too large for simple diffusion, and its poor miscibility with lipids, limiting its ability to cross the enterocytes of the small intestine (National Library of Medicine 2009) Micelles and liposomes as drug delivery systems offer several advantages, such as increasing drug half‐life, reducing toxicity, and improving efficacy (Qian et al. 2023). Romana et al. (2020) demonstrated that a liposomal/micellar carrier system could enhanced the bioavailability of poorly soluble drugs. Thangavelu et al. (2022) reported that liposomes significantly improved the pharmacological effects of gingerol in NSCLC induced BALB/c mice compared to gingerol alone. Our analysis revealed that PEG‐OA nanocarriers exhibit excellent dispersion, uniformity, symmetrical and systematic optical arrangement, low polydispersity, and spherical particle shape, suggesting they may indeed be micelles or liposomes. A comparison of AGS cell treatment with silibinin‐loaded micelle/liposome nanoparticles (SLNs) and silibinin alone showed that the lower dose of SLNs produced the same anticancer effects as the higher dose of silibinin alone. Thus, our designed nanoparticles can increase drug delivery into cancer cells without exhibiting cytotoxicity.

Dalimi‐Asl, Babaahmadi‐Rezaei, and Mohammadzadeh (2020) revealed that 250 μM of silibinin exhibited antiproliferation activities on MCF‐7 cells after 24 h. Zhang et al. (2018) demonstrated that the IC_50_ values of silibinin were 122.83, 201.69, and 69.09 μM, in AGS, BGC‐823, and SGC‐7901 cells, respectively. Hossainzadeh et al. (2019) reported that the IC_50_ of silibinin encapsulated in polymersome nanoparticles was 45.06 μg/mL in the MDA‐MB‐231 breast cancer cell line after 24 h. Pourgholi et al. (2021) showed that the IC_50_ of silibinin‐loaded polymeric nanoparticles was 55.23 and 48.21 μg/mL in MDA‐MB‐231 and MCF‐7 breast cancer cells, respectively. In this study, the IC_50_ value of silibinin loaded in nanoparticles for AGS gastric cancer cells was 28.21 μg/mL after 24 h, while the IC_50_ value of silibinin alone was 60.24 μg/mL. This demonstrates that micelles/liposomes nanoparticles enhanced silybinin delivery to AGS gastric cancer cells. According to the schematic image, micelles and liposomes first merge with the cell membrane and then release their content into the cells. Our results showed that different doses of SLNs (0–100 μg/mL) had < 5% cytotoxicity on HFF2 normal cells. Additionally, the cytotoxicity effect of micelles/liposomes nanoparticles was < 5% on AGS cancer cells. It appears that micelles/liposomes nanoparticles can enhance the anticancer and antiproliferative activities of silibinin.

Bioinformatic approaches have predicted that the majority of human genes be regulated by miRNAs. Zadeh et al. (2016) demonstrated the under‐expression of onco‐miRs miR‐21 and miR‐155, alongside the overexpression of putative target genes (*P53*, *BID*, and *CASP‐9*). Kumazaki et al. (2013) showed that resveratrol, an anticancer phytochemical, suppressed cell growth by upregulating the tumor‐suppressive miR‐34a, and downregulating its target gene *E2F3*. Safari et al. revealed that miR‐181a, acting as a tumor suppressor, was upregulated in LNCaP prostate cancer cells following green tea induction (Kumazaki et al. 2013). However, (Lu et al. 2018) reported that miR‐181a functions as an onco‐miR in gastric cancer cells. Hatami et al. found that quercetin‐loaded solid lipid nanoparticles inhibited cell migration, leading to a decrease in *β‐catenin* and Smad2/3 protein levels and an increase in *E‐cadherin* gene expression (Hatami et al. 2023). Poursani et al. showed that the copper chelating agent TEPA, acting as a copper trafficking blocker, reduced TGF‐β levels and lung metastasis in a TNBC mouse model. Additionally, TEPA significantly decreased TGF‐β signaling pathways, including TGF‐β/WNT/β‐catenin and TGF‐β/SMAD2&3 (Poursani et al. 2023).

In the study, we revealed the upregulation of miR‐34a with tumor suppressor function and the downregulation of miR‐181a, an onco‐miR following treatment with silibinin‐loaded nanoparticles (SLNs). Furthermore, our in silico analysis predicted that *TGFB*, *Smad3*, and *β‐catenin* (*CTNNB*) were putative targets of miR‐34a‐5p, while *E‐Cadherin* (*CDH1*) is a potential target of miR‐181a‐5p. Our quantitative measurements demonstrated the downregulation of migration‐related genes (*TGFB*, *Smad3*, and *β‐catenin*) and the upregulation of the antimigration *E‐Cadherin* gene in SLNs‐treated AGS cells. Additionally, the scratch test confirmed that silibinin‐loaded nanoparticles (SLNs) exhibited antimetastatic effects. The signaling pathways promoting cancer cell growth and migration have significant overlap (Stuelten, Parent, and Montell 2018). Therefore, it appears that silibinin can inhibit cancer cells through overlapping factors between cell proliferation and migration. Moreover, silibinin specifically affect cancer cell growth, migration and death by regulating miRNAs and their target genes.

## 5 Conclusions

This study demonstrated that PEG‐OA nanoparticles possess a spherical structure with low polydispersity and small dimensions (45–60 nm). Our finding suggest that micelles/liposomes nanostructures can effectively be used for delivering silibinin and other insoluble agents into cancerous cells. Furthermore y, silibinin‐loaded micelle/liposomes (SLNs), at low doses, were able to inhibit the proliferation and migration of AGS cancer cells while inducing apoptosis. One of the key functions of silibinin appears to be its ability to regulate cell proliferation, migration and death in AGS cancer cells through the modulation of miR‐181a, miR‐34a, and their potential targets.

## Author Contributions

**Mohsen Karami Fath:** conceptualization (equal), data curation (equal), investigation (equal), methodology (equal). **Parastoo Vakilinezami:** conceptualization (equal), data curation (equal), investigation (equal), methodology (equal). **Zohre Abdoli Keleshtery:** data curation (equal), investigation (equal). **Zahra Sima Azgomi:** investigation (equal), methodology (equal). **Sharareh Nezamivand Chegini:** data curation (equal), investigation (equal). **Mahdi Shahriarinour:** conceptualization (equal), formal analysis (equal), resources (equal), writing – review and editing (equal). **Saman Seyfizadeh Saraabestani:** data curation (equal), investigation (equal). **Mahzad Diyarkojouri:** investigation (equal), methodology (equal). **Mohammad Nikpassand:** conceptualization (equal), formal analysis (equal), resources (equal), software (equal), writing – original draft (equal), writing – review and editing (equal). **Najmeh Ranji:** conceptualization (equal), data curation (equal), software (equal), writing – original draft (equal), writing – review and editing (equal).

## Ethics Statement

This study was carried out in strict accordance with the recommendations of the ethics committee, and the study protocol was approved by the Ethics Committee of Human Experiments of Rasht Branch, Islamic Azad University, Rasht, Iran (IR.IAU.RASHT.REC.1401.040).

## Conflicts of Interest

The authors declare no conflicts of interest.

## Data Availability

Data available on request from the authors.
